# Gold nanoparticles attenuate albuminuria by inhibiting podocyte injury in a rat model of diabetic nephropathy

**DOI:** 10.1007/s13346-019-00675-6

**Published:** 2019-10-21

**Authors:** Ghada Alomari, Bahaa Al-Trad, Salehhuddin Hamdan, Alaa Aljabali, Mazhar Al-Zoubi, Nesreen Bataineh, Janti Qar, Murtaza M. Tambuwala

**Affiliations:** 1 Department of Bioscience, Faculty of Science, Universiti Teknologi Malaysia, 81310 Skudai, Johor Malaysia; 2 Department of Biological Sciences, Yarmouk University, Irbid, 21163 Jordan; 3 Faculty of Pharmacy, Department of Pharmaceutical Sciences, Yarmouk University, Irbid, Jordan; 4 Faculty of Medicine, Department of Basic Medical Sciences, Yarmouk University, Irbid, Jordan; 5 School of Pharmacy and Pharmaceutical Science, SAAD Centre for Pharmacy and Diabetes, Ulster University, Coleraine, County Londonderry Northern Ireland, UK

**Keywords:** Gold nanoparticles, Diabetic nephropathy, Streptozotocin, Rats, Nanomedicine

## Abstract

Several recent studies have reported that gold nanoparticles (AuNPs) attenuate hyperglycemia in diabetic animal models without any observed side effects. The present study was intended to provide insight into the effects of 50-nm AuNPs on diabetic kidney disease. Adult male rats were divided into three groups (*n* = 7/group): control (non-diabetic, ND), diabetic (D), and diabetic treated intraperitoneally with 50-nm AuNPs (AuNPs + D; 2.5 mg/kg/day) for 7 weeks. Diabetes was induced by a single-dose injection of 55 mg/kg streptozotocin. The result showed that AuNP treatment prevented diabetes-associated increases in the blood glucose level. Reduction in 24-h urinary albumin excretion rate, glomerular basement membrane thickness, foot process width, and renal oxidative stress markers was also demonstrated in the AuNP-treated group. In addition, the results showed downregulation effect of AuNPs in renal mRNA or protein expression of transforming growth factor β1 (TGF-β_1_), fibronectin, collagen IV, tumor necrosis factor-α (TNF-α), and vascular endothelial growth factor-A (VEGF-A). Moreover, the protein expression of nephrin and podocin, podocyte markers, in glomeruli was increased in the AuNPs + D group compared with the D group. These results provide evidence that 50-nm AuNPs can ameliorate renal damage in experimental models of diabetic nephropathy through improving the renal function and downregulating extracellular matrix protein accumulation, along with inhibiting renal oxidative stress and amelioration of podocyte injury.

## Introduction

Diabetic nephropathy (DN) is the major cause of end-stage renal failure and mortality in diabetic patients all over the world [1]. In DN, the first detected histological alteration usually appears on glomeruli as a thickening of the glomerular basement membrane (GBM) and tubular basement membrane (TBM) [2]. Both GBM thickening and glomerular hyperfiltration lead to the progression of microalbuminuria, while the leading cause for renal insufficiency in DN is the mesangium expansion, which develops at a later stage [3]. The expanded mesangium cells restrict and distort glomerular capillaries leading to the collapsing of glomerular capillaries and in turn reducing the filtration surface and decrease creatinine clearance [2, 4].

Mesangial expansion and GBM thickening occur as a result of increased expression and accumulation of the extracellular matrix (ECM) proteins such as collagen, fibronectin, and laminin in the mesangium and renal tubulointerstitium of the glomerulus and basement membranes [5]. This subsequently resulted from their increased production, decreased degradation, or both which eventually resulting in renal fibrosis and DN [2, 4–6]. Chronic hyperglycemia is the major initiator of these changes through increased oxidative stress, overproduction and action of advanced glycation end products (AGE), and activation of several growth factors, such as transforming growth factor-β1 (TGF-β_1_) and vascular endothelial growth factor (VEGF-A) [7].

Podocytes are highly specialized cells that act as size and charge barriers to protein and maintain the integrity of the glomerular filtration barrier [8]. One of the significant events in the pathogenesis of DN is the progressive podocyte injury, which expresses the recession of the podocyte foot processes that lead to defacement, loss of slit diaphragm proteins, detachment, and finally cell apoptosis [9]. Different studies have shown that the development of albuminuria and the acceleration of glomerular structural abnormalities in DN are associated with podocyte injury and loss [8].

Despite the available modern therapies that can delay the onset of DN (glucose and blood pressure control drugs), numerous patients continue to show progressive renal damage. Thus, it is extremely necessary to identify novel therapeutic strategies that could specifically target the progression of DN [10]. Recently, nanoparticles (NPs) have been widely used in biomedical applications because they can pass through biological barriers and their ability to enhance the bioavailability of therapeutic agents [11, 12]. Several studies have revealed that gold nanoparticles (AuNPs) exhibited anti-hyperglycemic, anti-inflammatory, and anti-oxidative activities in streptozotocin (STZ)-induced diabetic animal model [13, 14]. Hyperglycemia and oxidative stress are important factors in the development of diabetic microvascular complications including DN [15, 16]. Recent studies have demonstrated that pomegranate peel extract-stabilized 20-nm AuNPs and 13-nm naked AuNPs reduced STZ-induced renal fibrosis, inflammation, and oxidative stress [17, 18]. However, the maximized kidney targeting at the cellular (mesangium), tissue, and organ levels will be achieved via the usage of 50-nm AuNPs [19]. To the best of our knowledge, the effects of 50-nm AuNPs on the albuminuria, podocyte injury, oxidative stress, inflammation, and angiogenesis have not yet been studied in DN. The goals of the current study were to demonstrate the effects of 50-nm AuNPs in the STZ-induced diabetic rats and to explore their protective effects against diabetic albuminuria and podocyte injury.

## Materials and methods

### Materials

Sodium citrate trihydrate, HAuCl_4_, RNAlater solution, and Periodic Acid Schiff staining kit were all provided from Sigma-Aldrich, (St. Louis, USA). Streptozotocin and antibodies against TGF-β_1_ (Cat. No. sc-146) were both purchased from Santa Cruz biotechnology (Santa Cruz, USA). Albumin Rat ELISA kit, HRP/DAB IHC Detection Kit-Micropolymer, and the primary antibodies against Collagen IV (Cat. No. ab6586), Fibronectin (Cat. No.ab2413), nephrin (Cat. No.ab1830990), podocin (Cat.No.ab50339) were all provided from Abcam (Cambridge, UK). The superoxide dismutase assay kit was purchased from Cayman Chemical, (Ann Arbor, USA). Catalase assay kit was provided from Abbexa (Cambridge, UK). The Total RNA extraction kit was from iNtRON Biotechnology, Inc. (Seongnam, Korea). cDNA reverse transcription kit and SYBR green PCR master mix were purchased from Takara (Beijing, China). All primers were synthesized by Integrated DNA Technologies, INC. (Coralville, IA, USA). All other chemicals, buffers, and solvents used were of pure analytical grade.

### Synthesizing and characterization of AuNPs

The preparation and characterization of AuNPs were mentioned in our previous report [20]. In brief, 300 μl of an aqueous solution of 1% (w/v) HAuCl_4_ was added to 30 ml of ultra-pure H_2_O and brought to boil on magnetic hotplate (BT lab system, UK) while gently stirring at 150 rpm. Once the solution reaches boiling, 900 μl of freshly prepared aqueous of 1% (w/v) sodium citrate trihydrate was added to the reaction mixture. The reaction was allowed to proceed for 10 min where the AuNP formation was completed as indicated by the color transformation to ruby-red from yellow. Size and morphology of the resulted AuNPs were characterized using a scanning electron microscope (Quanta FEG SEM, Thermo Fisher Scientific, UK), dynamic light scattering (DynaPro Titan Wyatt Technology Corporation) equipped with a laser wavelength of *λ* = 830 nm, scattering angle of 2° equipped with Dynamics software version 6.9.2.11, and zeta potential instruments were recorded on (Malvern Instruments Zetasizer-Nano ZS) in which 0.2 mg was suspended in 1 ml of 0.1 M sodium phosphate buffer pH 7.0. Zeta cells were pre-equilibrated at 21 °C with the buffer, and data were presented from 3 independent synthesis.

### Induction of diabetes and experimental protocols

All animal experimental procedures were carried out in accordance with the National Institutes of Health guidelines for the care and use of laboratory animals and approved by the committee of animal ethics at Yarmouk University (permission number: YU-20/12/2017). Adult male Wistar rats weighting 250 to 300 g were maintained under specific pathogen-free conditions at the animal house unit at Yarmouk University. After 1 week of adaptation, diabetes was induced by a single intraperitoneal injection of streptozotocin (STZ) (55 mg/kg; following overnight fasting). Three days after STZ injection, rats with a blood glucose level over 250 mg/dl were considered diabetic. Rats were divided into three groups (*n* = 7/group): control (non-diabetic, ND), diabetic (D), and diabetic treated intraperitoneally with 50 nm AuNPs (AuNPs + D; 2.5 mg/kg) daily for 7 weeks.

The size, dose, and the route of administration of AuNPs used in the present study were selected based on the previous study that reported that intraperitoneal administration of spherical, 50-nm AuNPs showed anti-hyperglycemia and anti-oxidative effect in STZ-induced diabetic mice without toxic effect over the vital organs [13].

### Urine, blood, and tissue collection

At the end of 7 weeks of treatment, the rats were placed in metabolic cages 1 day before sacrifice, and urine was collected for 24 h for the analysis of urine albumin concentration and the urine output. Then, the animals were weighed and lightly anesthetized with ethyl ether, and blood samples were collected (via cardiac puncture). The animals were sacrificed using an overdose of ethyl ether. The right kidney was removed and then transferred into RNAlater solution for the real-time PCR analysis. The left kidney was fixed with 4% paraformaldehyde for morphological and immunohistochemical analysis.

### Measurements of blood glucose, 24-h urinary albumin excretion rate, and renal oxidative stress markers

The blood glucose level was determined by glucometer (Accu-Chek Performa, Roche Diagnostics). Urine samples were centrifuged at 4 °C and 2000 rpm for 10 min. The urinary albumin in the supernatant was measured using Albumin Rat ELISA kit according to the manufacturer’s protocols.

Kidney tissues were homogenized in protein extraction buffer containing PBS and protease inhibitor. Commercially available kits were used according to the manufacturer protocols to identify the activities of superoxide dismutase and catalase. The renal malondialdehyde (MDA) level, a marker of lipid peroxidation, was assessed by a method described previously [21]. In brief, renal tissue homogenate (0.2 mg protein/ml) was combined with 2.0 ml of thiobarbituric acid (TBA), trichloroacetic acid (TCA), and TCA-TBA-HCI and was mixed thoroughly. Then, the solution was heated for 15 min in a boiling water bath, and after cooling, the flocculent precipitate was removed by centrifugation at 1000*g* for 10 min. The absorbance of the sample was determined at 535 nm against a blank that contains all the reagents minus the lipid.

### Morphology studies

After the fixation in 4% paraformaldehyde, the tissues were dehydrated in ascending series of alcohol, cleared in xylene, embedded in paraffin, sectioned at 4 μm, and stained with hematoxylin and eosin (H&E) and periodic acid-Schiff. For electron microscopy study, renal cortical tissues were cut into 1-mm^3^ cubes, fixed in 2.5% glutaraldehyde dissolved in 0.1 M sodium cacodylate buffer (v/v%) (pH 7.4) for 1 h, and then washed in the same buffer. The tissue fragments were post-fixed in sodium cacodylate-buffered 1% OsO_4_ (v/v%) for 2 h, dehydrated, and embedded in Spurr’s resin. Ultra-thin sections were cut using ultra-microtome (Reichert-Jung, Austria), contrasted with 4% uranyl acetate for 45 min, and subsequently incubated with lead citrate for 4 min at room temperature. Sections were examined under a Hitachi electron microscope (HT7700-Tokyo). The GBM thickness was measured and calculated as previously detailed [22]. The degree of podocyte detachment was calculated as the mean of foot process width (FPW). From each photograph, the arithmetic mean of the FPW was calculated according to the equation below [23]:

$$ \mathrm{FPW}=\frac{\pi }{4}\times \frac{\sum \mathrm{GBM}\ \mathrm{length}}{\sum \mathrm{foot}\ \mathrm{process}} $$

where Ʃ GBM length is the total GBM length measured in each photo and Ʃ foot process is the total number of foot processes counted in that photo. To correct for supposed random difference in the angle of section relative to the long axis of the podocyte, the correction factor of *π* /4 was used. GBM length was calculated by computerized measurement using the ImageJ software. Podocyte foot processes number was computed by hand [23].

### Immunohistochemistry

Immunohistochemical staining was performed with mouse and rabbit-specific HRP/DAB IHC Detection Kit-Micropolymer, according to the manufacturer’s protocol using specific primary antibodies against TGF-β_1_, ECM proteins, and podocyte markers as follows: collagen IV (1:100), fibronectin (1:50), nephrin (1:100), podocin (1:50), and TGF-β_1_ (1:100). Negative controls were run by replacing the primary antibody with PBS. Sections were examined under an Optika microscope and evaluated using Fiji ImageJ software.

### Real-time PCR

Total RNA was extracted from RNAlater-preserved kidney tissues using Total RNA extraction kit according to the manufacturer’s protocols. RNA quantity was determined using QuantiFluor RNA System (Promega, Madison, USA) and Quantus Fluorometer (Promega, Madison, USA). A cDNA reverse transcription kit was used to prepare the cDNA template, according to the manufacturer’s instructions. Quantitative real-time PCR was conducted using LineGene 9600 Real-Time PCR system (Bioer Technology Co., Binjiang, China), with the SYBR green PCR master mix, at 95 °C for 2 min and 45 cycles of 95 °C for 30 s, and 60 °C for 30 s. GAPDH was used as a non-regulated reference gene. PCR primers of TGF-β_1_, VEGF-A, collagen IV, fibronectin, and TNF-α primers were designed using Primer3 (http://www.ncbi.nlm.nih.gov/tools/primer-blast/) software and synthesized by IDT (Table 1). To ensure the specificity of the primers, a gel was run, in which a single band of expected size was obtained. The fold changes in the mRNA expression were determined using the 2^−ΔΔCT^ method [24].

**Table 1:** Sequences of primers used for quantitative real-time PCR

Gene^1^	Forward (5–3)	Reverse (5–3)
GAPDH	ATG GTG AAG GTC GGT GTG	GAA CTT GCC GTG GGT AGA
TGF-β_1_	GTG GAG CAA CAC GTA GAA C	TCC TTG GTT CAG CCA CT
VEGF-A	CGA ACA GAG AGA GGG ACA GG	GTC TGT CTG TCT GTC CGT CA
Fibronectin	TGA CCG ACG CTA CAG AAA CT	TTG AGC GTG TAC AGG TGG AT
TNF-α	TTC GGA ACT CAC TGG ATC CC	GGA ACA GTC TGG GAA GCT CT
Collagen IV	TTG GCT TTC CTG GTA GTC GT	CAA CCT TTC CTG CTT CAC CC

^1^*GAPDH*, glyceraldehyde 3-phosphate dehydrogenase; *TGF-β*_*1*_, transforming growth factor-β1; *VEGF-A*, vascular endothelial growth factor-A; *TNF-α*, tumor necrosis factor-α

### Statistical analysis

All data are expressed as the mean ± SEM; the minimum number of replicates used in the analysis was 6. Differences between groups were calculated by one-way analysis of variance (ANOVA) using SPSS software (SPSS Inc., Chicago, IL). The significant value of difference was considered when the *P* value < 0.05.

## Results

### Characterization of AuNPs

Characterization of the generated particles revealed that the generated AuNPs mainly consist of spherical particles with an average hydrodynamic diameter of 51.8 ± 0.7 nm and with a polydispersity index of 0.26%. Furthermore, the surface zeta potential related to the surface charge was − 40.0 ± 0.2 [20].

### Effect of AuNPs on metabolic and biochemical parameters

After 7 weeks of STZ administration, diabetic animals showed significant increases in the blood glucose level, kidney weight/body weight ratio, and 24-h urinary albumin excretion rate, indicating that we successfully established an experimental animal model of type 1 diabetes possessing DN (*P* < 0.05; Table 2). Treatment with AuNPs did not affect the kidney weight/body weight ratio in diabetic rats significantly. However, the AuNP treatment significantly decreased blood glucose level and 24-h urinary albumin excretion rate compared with the D group (*P* < 0.05; Table 2).

**Table 2:** Effect of AuNPs on metabolic and biochemical parameters

Parameters	ND	D		AuNPs + D	
		Mean ± SEM	*P* value*	Mean ± SEM	*P* value^$^
Blood glucose (mg/dl)	113.1 ± 5.6	340.83 ± 42.11*	< 0.0001	144.1 ± 25.48^$^	< 0.0001
Kidney weight/body weight ratio	0.60 ± 0.02	1.12 ± 0.02*	< 0.0001	1.035 ± 0.085	0.1992
Urinary albumin excretion rate (mg/day)	0.14 ± 0.04	0.68 ± 0.06*	< 0.0001	0.32 ± 0.07^$^	0.0004

Data represent the mean ± SEM. *P value compared with the ND group. ^$^*P* value compared with D. *ND*, non-diabetic; *D*, diabetic; *AuNPs*, gold nanoparticles

### Renal histopathological and ultrastructural changes

**Fig. 1:**
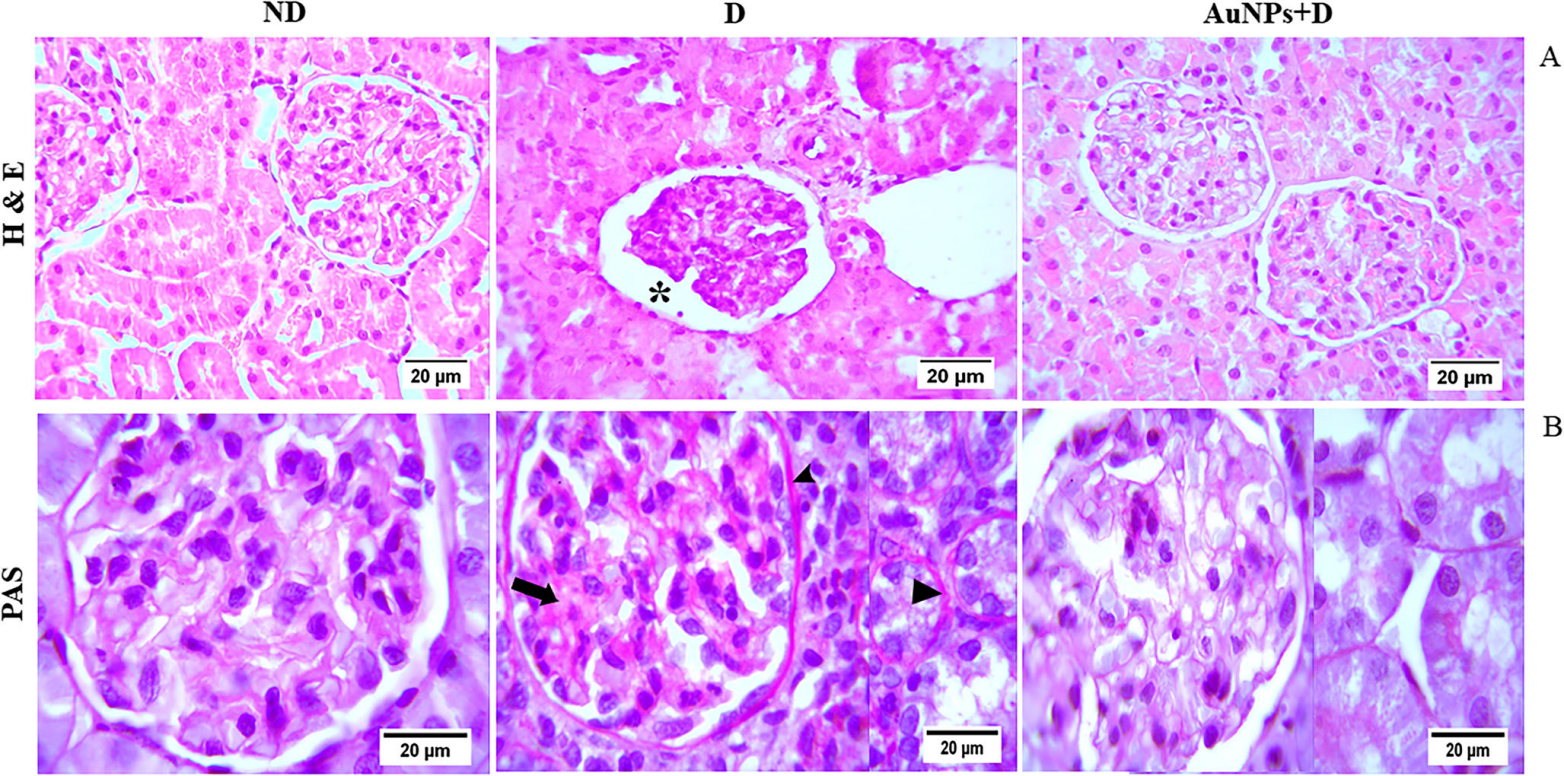
**a** H&E-stained sections (magnification, × 400) show intact and organized structure glomerulus in the ND group. Disorganized architecture of glomerulus in the D group and normal glomeruli the AuNPs + D group. Asterisk indicates expansion in urinary space. **b** PAS-stained sections (magnification, × 1000) show that the percentage of the PAS-positive material, indicative of increased fibrosis, was significantly increased in the D group compared with the ND group. Arrow head: basement membrane thickening, arrow: mesangial expansion. AuNP treatment inhibited the increase in the PAS-positive material in the AuNPs + D group

The histological morphology showed organized intact glomeruli, Bowman’s capsule, and tubular structure in the ND group (Fig. 1a). In contrast, the histological examination of the renal tissue in the D group showed disorganized architecture characterized by expansion in urinary space and closure of capillary loops and tubular atrophy and increased vacuolization of renal tubular epithelial cells. However, AuNP treatment ameliorates these abnormal histological findings in the AuNPs + D group. Light microscopy sections of PAS staining showed mesangial expansion and thick basement membranes in the D group compared with the ND group) Fig. 1b). While in AuNPs + D group, GBM showed normal thickness with decreased in mesangial matrix deposition.

**Fig. 2:**
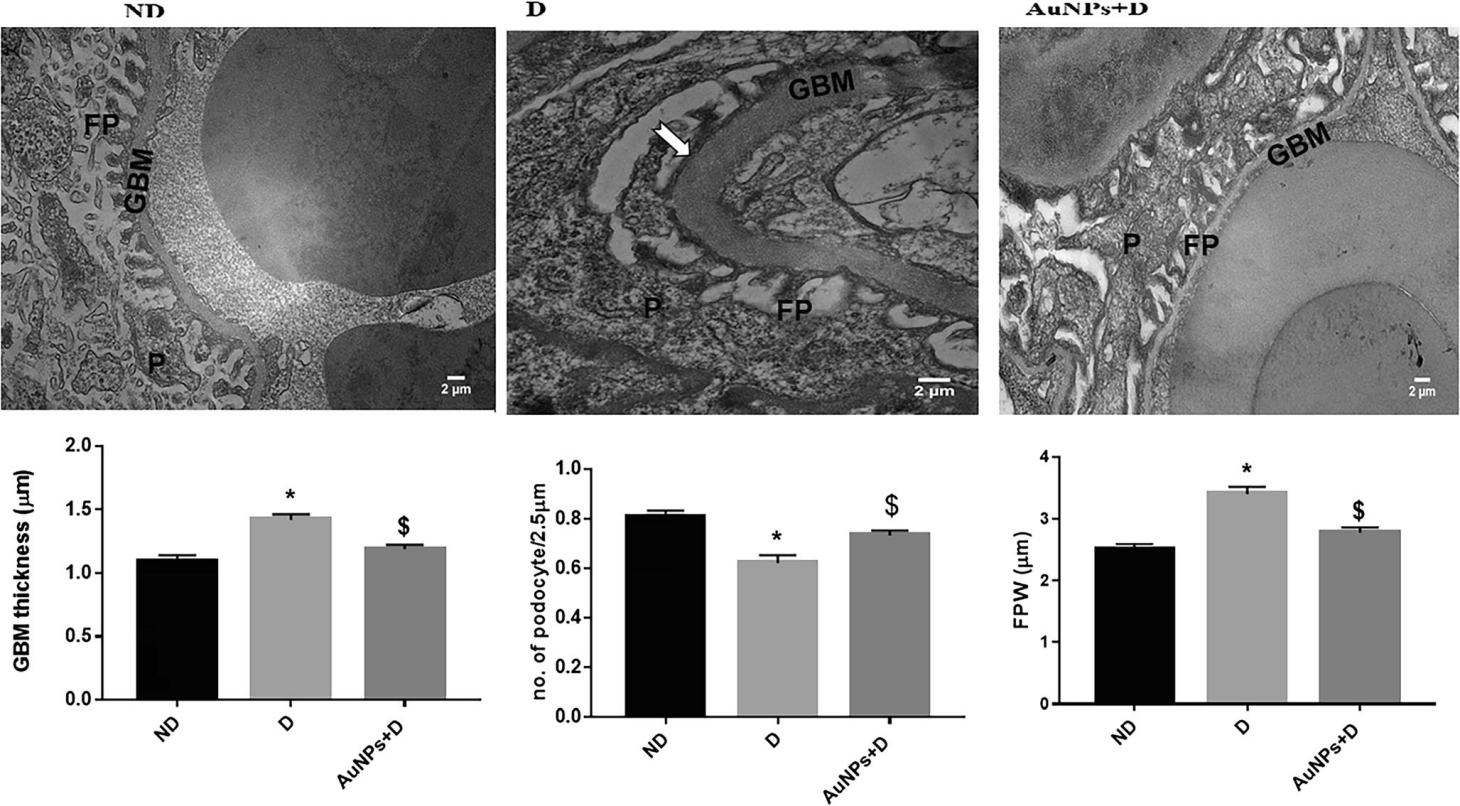
Glomerular ultrastructure in the different treated groups. **a** Representative electron microscopy images of podocyte foot processes and the GBM (magnification: ND and AuNPs + D × 6000, D × 8000). White arrow: foot process effacement. **b** Glomeruli were evaluated to quantify the GBM thickness. *P < 0.05 compared with the ND group. ^$^*P* < 0.0001 compared with D, number of podocyte. *P < 0.05 compared with the ND group. ^$^*P* < 0.0025 compared with D and FPW. *P < 0.05 compared with the ND group. ^$^*P* < 0.0001 compared with D. Data represent the mean ± SEM. ND, non-diabetic; D, diabetic; AuNPs, gold nanoparticles; GBM, glomerular basement membrane thickness; FPW, foot process width; P, podocyte

Several ultrastructural abnormalities were present in the electron microscopy sections (Fig. 2) in the D group such as diffused thickening of the GBM, a reduction in the podocyte number, and diffused foot process effacement, along with a reduced podocyte per glomerulus ratio. Treatment of diabetic rats with AuNPs ameliorated the above pathogenic findings.

### Effect of AuNPs on renal oxidative stress markers

Diabetic animals showed significant decreases in the renal tissue SOD and catalase activities and increase in the MDA level compared with the ND group (*P* < 0.05; Table 3). Notably, AuNP administration restored the renal SOD and catalase activity and the MDA level to the normal values.

**Table 3:** Effect of AuNPs on renal oxidative stress markers

Parameters	ND	D		AuNPs + D	
		Mean ± SEM	*P* value*	Mean ± SEM	*P* value^$^
SOD (U/mg)	2.84 ± 0.14	2.07 ± 0.36^*^	0.043	2.90 ± 0.20^$^	0.046
Catalase (U/mg)	5.54 ± 0.89	1.67 ± 0.31^*^	0.015	4.82 ± 1.29^$^	0.035
MDA (μmol/mg)	0.12 ± 0.04	0.49 ± 0.17^*^	0.014	0.17 ± 0.045^$^	0.028

Data represent the mean ± SEM. *P value compared with the ND group. ^$^*P* value compared with D. *ND*, non-diabetic; *D*, diabetic; *AuNPs*, gold nanoparticles; *SOD*, superoxide dismutase; *MDA*, malondialdehyde

### Effect of AuNPs on the renal expression of fibrosis, angiogenesis, and inflammation markers

**Fig. 3:**
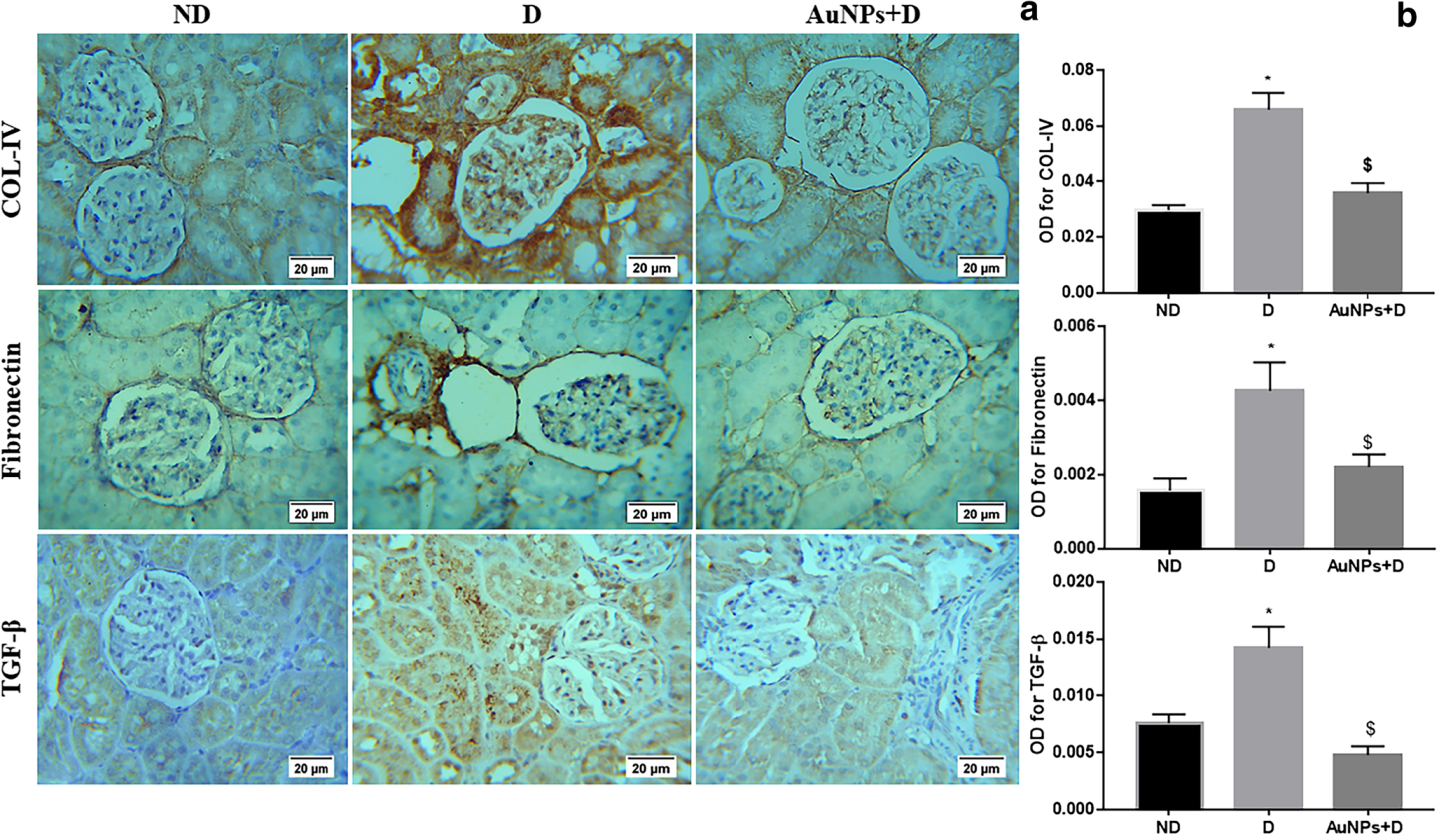
Immunohistochemistry detects that AuNP treatment inhibits the collagen IV, fibronectin, and TGF-β_1_ expression in the diabetic kidney. **a** Immunohistochemical stain of the kidney sections (magnification, × 400) shows that the immunostaining (brown staining) in the glomeruli was much stronger in the D group compared with the ND group. AuNP treatment inhibited the increase in the immunostaining in the AuNPs + D group. **b** Immunohistochemistry optical density score. Data represent the mean ± SEM. (COL-IV *P < 0.05 compared with the ND group, ^$^*P* < 0.0001 compared with D. Fibronectin *P < 0.05 compared with the ND group, ^$^*P* = 0.0074 compared with D. TGF-β_1_ *P < 0.05 compared with the ND group, ^$^*P* < 0.0001 compared with D). ND, non-diabetic; D, diabetic; AuNPs, gold nanoparticles; TGF-β_1_, transforming growth factor-β_1_

**Fig. 4:**
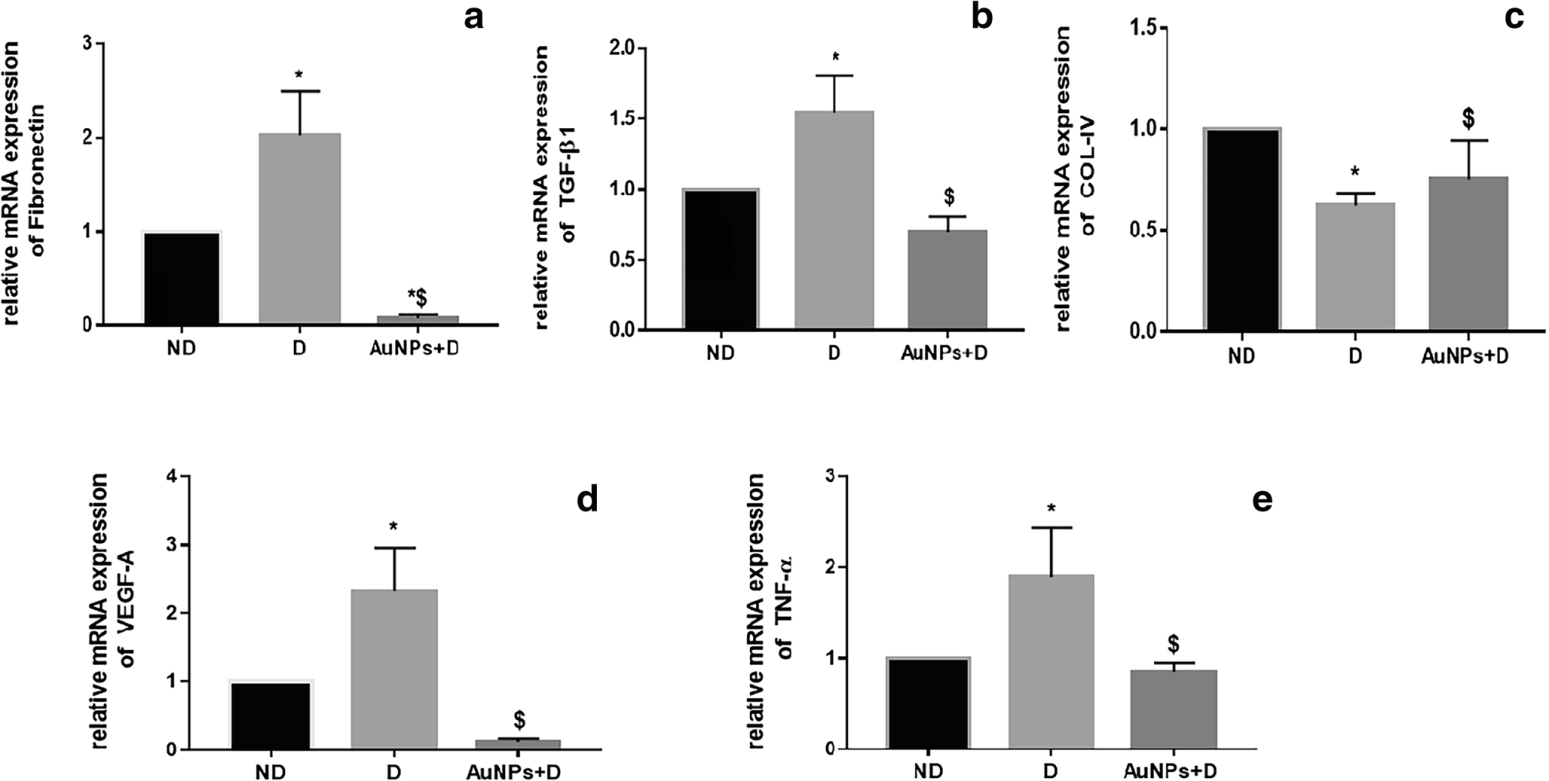
Effect of AuNP treatment on the mRNA expression in the normal and diabetic kidney. **a** Fibronectin, *P < 0.05 compared with the ND group; ^$^*P* < 0.0001 compared with D. **b** TGF-β_1_, *P < 0.05 compared with the ND group; ^$^*P* = 0.0008 compared with D. **c** Collagen IV, *P < 0.05 compared with the ND group; ^$^*P* = 0.15 compared with D. **d** VEGF-A, *P < 0.05 compared with the ND group; ^$^*P* = 0.0006 compared with D. **e** TNF-α; *P < 0.05 compared with the ND group; ^$^*P* = 0.0082 compared to D. Data represent the mean ± SEM. ND, non-diabetic; D, diabetic; AuNPs, gold nanoparticles; TGF-β_1_, transforming growth factor-β_1_; VEGF-A, vascular endothelial growth factor-A; TNF-α, tumor necrosis factor-α

The immunohistochemistry analysis demonstrated that diabetes was associated with an increase in the intensity of immunostaining for collagen IV, fibronectin, and TGF-β_1_ (Fig. 3a, b), which are the major ECM proteins that lead to the development of the mesangial matrix expansion in the status of DN [25]. Consistent with the immunohistochemical findings, the levels of mRNA encoding for fibronectin and TGF-β_1_ were significantly greater in the D than in the ND group (Fig. 4a, b; *P* < 0.05). However, the collagen IV mRNA levels did not differ significantly between D and AuNPs + D groups (Fig. 4c; *P* > 0.05). The treatment of diabetic rats with AuNPs downregulates the expression of these fibrosis markers, suggesting that AuNP treatment inhibits the progression of renal fibrosis in the AuNPs + D group.

Upregulation in the expression of VEGF-A, an angiogenic factor, and TNF-α, an inflammatory marker is often observed in the DN. Figure 4 d and e show that the mRNA expression levels of VEGF-A and TNF-α were significantly increased in the D group compared with the ND group (*P* < 0.05). However, the treatment with AuNPs downregulates the expression of VEGF-A and TNF-α in the AuNPs + D group.

### Effects of AuNP treatment on the expression of podocyte markers (nephrin and podocin)

**Fig. 5:**
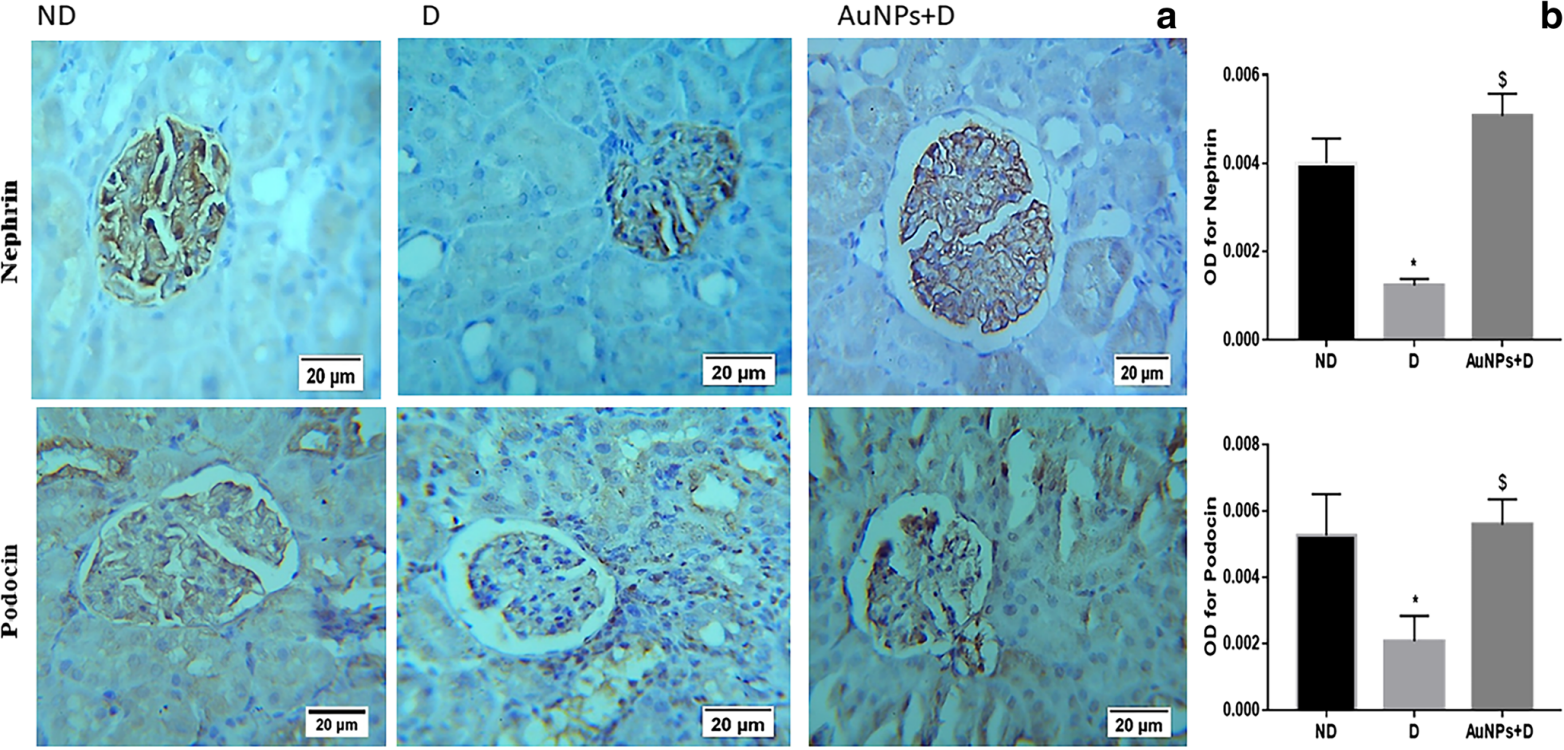
Immunohistochemistry detects that AuNP treatment restores the nephrin and podocin expression in the diabetic kidney. **a** Immunohistochemical stain of the kidney sections (magnification, × 400) shows that the immunostaining (brown staining) in the glomeruli was much stronger in the ND group compared with the D group. AuNP treatment inhibited the decrease in the nephrin and podocin immunostaining in the AuNPs + D group. **b** Immunohistochemistry optical density score. Data represent the mean ± SEM. (Nephrin; *P < 0.05 compared with the ND group. ^$^*P* < 0.0001 compared with D. Podocin; *P < 0.05 compared with the ND group. ^$^*P* = 0.0006 compared with D). ND, non-diabetic; D, diabetic; AuNPs, gold nanoparticles

The expression intensity and the distribution pattern of nephrin and podocin in the glomeruli were assessed by immunohistochemical staining (Fig. 5a). In the D group, the expression intensity of nephrin and podocin were significantly decreased compared with the ND group (Fig. 5b, *P* < 0.05). However, AuNPs fully improved the immunohistochemical staining intensity of both podocyte markers in the AuNPs + D group.

## Discussion

Due to their unique physical and chemical properties, many pieces of research have been conducted to elucidate their potential efficiency in the treatments of many diseases. Accordingly, using AuNPs as a promising antidiabetic agent just started to emerge [13, 26–28]. To explore the effect of AuNPs on the DN, an experimental DN similar to DN in type 1 diabetic patient was established successfully in this study that characterized by hyperglycemia and albuminuria that were associated with the loss of podocytes, effacement of foot process, and thickening of the GBM and glomerulosclerosis [2]. Our results revealed that 50-nm AuNPs effectively ameliorated DN in a rat model of STZ-induced type 1 diabetes.

Podocytes play a major role in maintaining the integrity and permeability of the glomerular filtration barrier [8]. In DN, a reduction in podocyte number, the effacement of podocyte foot processes, and the loss of slit diaphragm proteins such as nephrin and podocin result in a leakage of albumin and proteinuria [29]. The results of the present study showed that the levels of urinary albumin were significantly increased in diabetic rats which were associated with the loss of podocytes, widening and effacement of foot process, and decrease in the staining intensity of nephrin and podocin. Our data indicated that AuNPs significantly reduced albuminuria and the podocyte ultrastructural abnormalities and restored the staining intensity of nephrin and podocin, suggesting a protective effect for AuNPs on the structure of podocytes in the diabetic conditions.

In addition, GBM and TBM thickening along with mesangial expansion seem to be a critical determining factors for the progress of proteinuria and the advance of kidney dysfunction in DN [30]. Both mesangial expansion and GBM and TBM thickening are a consequence of ECM protein accumulation due to their increased production, decreased degradation, or both [2]. Our results showed that the GBM thickening was significantly lower in the AuNPs + D rats compared with the D group. In addition, the upregulation of tissue collagen and fibronectin, the major constituent of ECM proteins, in the diabetic kidney is effectively reversed after the AuNP treatment. Many studies support a role for hyperglycemia in the increased synthesis of many ECM proteins that accumulate within the mesangium and GBM of diabetic kidneys resulting in changes in permeability of the filtration barrier [31]. Improved glycemic control in patients with diabetes is, therefore, a goal in the attempt to slow the rate of progression of diabetic renal disease [32]. ECM accumulation in diabetes is induced by TGF-β_1_, which enhances collagen, fibronectin, and laminin synthesis and inhibits proteins that mediate the ECM protein degradation [33]. Studies have shown that high glucose environment could increase TGF-β_1_ expression in renal tubular epithelial cells and activate ECM protein deposition in glomeruli [34]. Therefore, the TGF-β_1_ pathway is a therapeutic target for DN [35]. In this study, TGF-β_1_ mRNA and protein levels were increased in the D group and reduced in the AuNP-treated rats, which is in turn reduce the accumulation of the ECM proteins, collagen IV, and fibronectin. This may provide insight into the mechanism whereby AuNP contributes to reduced mesangial matrix expansion in the diabetic condition. Consistent with our results, AuNPs significantly decreased the expression of fibronectin and TGF-β_1_ and inhibit matrix deposition in pancreatic ductal adenocarcinoma [36]. The exogenous administration of AuNPs is documented to afford protection against liver fibrosis [37]. In addition, gold nanorods modulate cell-mediated matrix remodeling and decrease the mRNA expression of type I collagen in neonatal rat cardiac fibroblasts [38].

Oxidative stress, defined as an imbalance in the oxidant/antioxidant production, has been considered to be one of the primary factors for the development of DN [15, 16]. Excessive production of reactive oxygen species (ROS) in multiple cell types including mesangial cells and podocytes activates various protein kinases, cytokines, and transcription factors which eventually cause increased expression of ECM proteins and progression to renal fibrosis [31, 39]. Thus, neutralizing the ROS induced by hyperglycemia is effective in preventing experimentally induced diabetes and its complications such as DN [40]. SOD and catalase are considered the key antioxidant enzymes which detoxify ROS to water in the kidney [1]. In our study, AuNP treatment significantly reduced the renal oxidative stress by increasing the activities of renal SOD and catalase and by reducing the tissue MDA level. These results were in agreement with previous in vivo studies that showed that AuNPs have anti-oxidative and anti-hyperglycemic activities in diabetic animal models [13, 41].

Several studies have shown the crucial role of kidney inflammation in promoting the development and progression of DN [42–44]. In particular, inflammatory cytokines have emerged as being closely involved in the pathogenesis of DN [45]. For instance, TNF-α has been reported as one of the major inflammatory cytokines that is believed to play an important role in the pathogenesis of DN. TNF-α has been reported to be responsible for the production of ROS, induction of cell injury, and increases in endothelial permeability [44, 45]. In the present study, AuNPs were found to be associated with the suppression of the inflammation in the AuNPs + D group. Specifically, treatment with AuNPs showed a significant reduction in the mRNA expression levels of TNF-α down to the normal levels as compared with the D group, indicating the effect of AuNPs on suppressing the inflammation through preventing TNF-α over expression. This result is consistent with other studies which described AuNP as an anti-inflammatory agent in different diseases including diabetes [14, 46–48].

Moreover, vascular endothelial growth factor (VEGF) is the most potent angiogenic factor and its upregulation often is observed in many pathologic conditions including DN [49]. Upregulated VEGF synthesis is accompanied by the glomerular hypertrophy and urinary albumin excretion in the DN [10, 49, 50]. Several studies have indicated that VEGF mRNA, protein expression, and activity are enhanced in kidneys with early DN [51–54]. In the current study, the level of VEGF-A was significantly increased in the kidney of diabetic rats. However, AuNP treatment successfully reversed the increase in the expression of the VEGF in the AuNPs + D group. Our findings underscored the role of AuNPs as anti-angiogenic treatment, which is consistent with previous findings [55]. Nevertheless, the mechanism of action of the AuNPs as anti-angiogenic factor is not fully understood; however, previous report proposed that the AuNPs inhibit the activity of heparin-binding growth factors (HB-GFs) such as vascular endothelial growth factor 165 (VEGF165) and basic fibroblast growth factor (bFGF) by the change in HB-GF conformation/configuration (denaturation) by the AuNPs [55].

Finally, due to the variability of parameters such as the size and surface charge of the particle, cell type, and dosing parameters, no straightforward deduction has arisen from the available studies regarding AuNP toxicity. However, a great advantage of AuNPs over other nanoparticles is that they show no cytotoxicity in human cells [56]. In the current study, daily administration of 50-nm AuNPs for 7 weeks was well tolerated by the animals (no significant changes in the feed intake, mortality rate, and behavior). In addition, the analysis of hepatic function showed no significant effects of 50-nm AuNP treatment on the serum aspartate aminotransferase (AST) and alanine aminotransferase (ALT) levels (data not shown).

## Conclusion

The results of this study demonstrated a potential curing efficiency of AuNPs on DN. The study outcomes confirmed that STZ treatment clearly raised the blood glucose level which enhanced oxidative stress and inflammation. The adverse effects of these outcomes were evident in renal tissue indicated by the accumulation of ECM proteins, GBM thickening, and podocyte injury which eventually lead to albuminuria. In contrast, AuNPs were able to show promising effects in preventing the adverse effects of hyperglycemia in renal tissue by preventing the accumulation of ECM proteins and the amelioration of podocyte injury through the downregulation of growth factors, inflammatory, and angiogenesis markers. In summary, the current study proposed a potential use of AuNPs for the treatment and prevention of diabetic nephropathy complications; however, future functional studies are required to understand the exact molecular mechanism of action at cellular and physiological levels.
